# Suitability of Engineering-Geological Environment on the Basis of Its Permeability Coefficient: Four Case Studies of Fine-Grained Soils

**DOI:** 10.3390/ma14216411

**Published:** 2021-10-26

**Authors:** Marian Marschalko, Zofia Zięba, Dominik Niemiec, David Neuman, Jakub Mońka, Jolanta Dąbrowska

**Affiliations:** 1 Institute of Geological Engineering, Faculty of Mining and Geology, VŠB-Technical University of Ostrava, 708 33 Ostrava, Czech Republic; marian.marschalko@vsb.cz (M.M.); dominik.niemiec@vsb.cz (D.N.); david.neuman.st@vsb.cz (D.N.); 2 Department of Civil Engineering, Faculty of Environmental Engineering and Geodesy, Wrocław University of Environmental and Life Sciences, 50-365 Wrocław, Poland; jakub.monka@upwr.edu.pl (J.M.); jolanta.dabrowska@upwr.edu.pl (J.D.)

**Keywords:** engineering geology, soil permeability, fine-grained soils, soil microstructure, methods of permeability coefficient determination, scanning electron microscope technique, Kozeny-Carman Formula, Slichter Formula, Seelheim Formula, laboratory soil permeability testing

## Abstract

The aim of the article is to compare two classifications systems of engineering-geological environment sustainability in terms of its permeability evaluated on the basis of permeability coefficient. The first evaluated classification assumes a permeable environment to be a positive characteristic in the engineering-geological assessment, while the other considers an impermeable environment as favourable. The four fine-grained soil materials were selected, as they had very similar, almost identical grains-size distribution, but different microstructure characterized by grains sphericity, angularity, and roughness. At the same time, the influence of changes in the density of soil materials (density index 10%, 30%, 60%, 90%) was analysed. Permeability coefficient was determined using six methods (empirical formulae, laboratory and microscopic analysis). The laboratory method falling head test (FHT) was taken as a reference test that reflected the actual water flow through the soil. It was found that with an increase in grain angularity and roughness (and a decrease in sphericity), the permeability coefficient was decreasing and this trend culminated along with gradual compaction. Moreover, the research shows that unsuitable methods may classify soil materials into wrong engineering-geological permeability classes, which may have negative consequences during engineering-geological or geotechnical assessment and cause subsequent problems in foundation engineering.

## 1. Introduction

An important factor in engineering geology is the permeability of the geological environment [1,2,3,4,5,6,7] which subsequently influences a number of boundary conditions in foundation engineering. Thus, it is vital to appropriately determine the most important parameter in the quantification of geological environment permeability, namely the permeability coefficient [8,9]. Due attention must be paid to the choice of methods in its determination [10,11,12,13,14] as well as the differences in the values obtained by different methods.

There are many methods of determining the permeability coefficient, but they may be generally divided into in-situ tests (i.e., pumping and borehole permeability tests), laboratory tests (i.e., constant water head test, falling water head test, capillary permeability test), empirical and predictive methods. Each of these groups has its advantages and disadvantages. In-situ tests return the most reliable results, however they are costly and technically difficult—erroneous results may be influenced by incomplete recognition of the geological structure of the layer. In laboratory tests, the challenge lies in preparing representative samples for testing, and sample size is limited [15,16]. Empirical formulae, which are widely used, are based mainly on the grain-size of soils and thus their use, although easy and quick, is subject to significant errors. Often these formulae, based only on grain-size diameters, do not take into account the relationship between porosity, compaction, specific surface area and permeability coefficient [17,18,19,20,21,22]. 

This problem is addressed in the article via demonstrating the differences based on the analysis of four soil materials of more or less identical grain-size distribution (grain size), but varying microstructure. The differences obtained in the permeability coefficient values mean that the permeability of the geological environment may not be determined correctly. A serious problem of engineering-geological investigations is the fact that the permeability coefficient is often determined based on empirical formulae, but not on more suitable laboratory, microscopic or in-situ methods. However, this may have severe consequences in the field of engineering geology, geotechnics, and foundation engineering. Wrong verdicts may often lead to inappropriate engineering design decisions, wrong redevelopment or foundation engineering projects, which is a negative phenomenon with a number of technical, economic and safety impacts.

The permeability of the geological environment influences many engineering structures, especially these where the presence of water may have fatal consequences, such as water reservoirs, dams, or other water management structures. The permeability of the geological environment has been investigated by a number of authors, and the most common criterion was the character of the engineering structure. For example, Chen et al. [23], Stark et al. [24], and Xu et al. [25] investigated the permeability of the geological environment in dams, Masset and Loew [26] studied underground structures and tunnels, and studies on the redevelopment of contaminated geological environment have been frequent [27].

In case of water flow through the soil, its microstructure reflecting the particle shape and their roughness is also very significant. It determines the capability to retain bound water as well as water movement [28]. Although many researchers have proved that the geometrical properties of soil particles determine the parameters and behaviour of subsoil [28,29,30,31], these findings are often neglected in engineering practice. 

The aim of the article was to determine the soil permeability coefficient of four fine-grained soils, taking into account their particle shape properties such as sphericity, angularity, and roughness under various density conditions. Apart from studying the effect of grains’ surface on the permeability coefficient, we also assessed different methods in determining the permeability coefficient, namely in terms of different purpose classifications of engineering environment permeability to determine suitability or unsuitability of a particular project. We assumed that the determination of permeability coefficient is influenced by the very method, but also by the permeability classification system, because it determines the relevant limits of the characteristic. It has certain consequences during the implementation of an engineering structure, where permeability plays a significant role. 

The study has important implications for engineering geology, geotechnics, foundation and civil engineering, as it demonstrated substantial differences in the determination of the permeability coefficient depending on the methods used to meet the boundary conditions of fine-grained soils. Using microscopic techniques and laboratory methods, much more accurate determination of permeability coefficient was obtained than in the empirical approaches.

## 2. Materials and Methods

### 2.1. Characteristics of the Soil Materials

**Figure 1:**
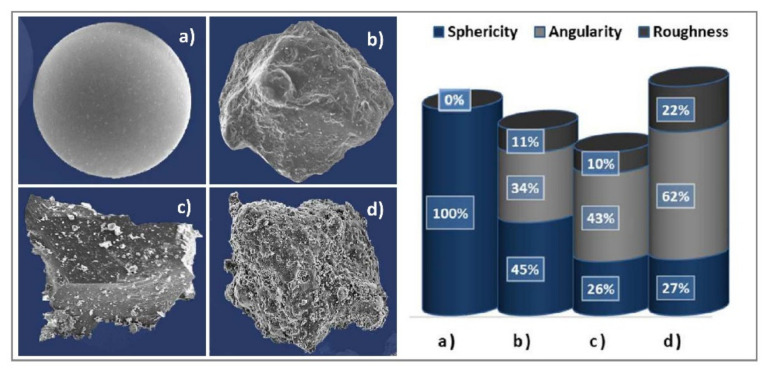
SEM images of 4 soil particles’ shapes: (**a**) glass microbeads (GM); (**b**) sandy silt from Krakowiany (SK); (**c**) sandy silt from Graniczna (SG); (**d**) fly ash (FA).

In the research, we determined the permeability coefficient by four case studies of fine-grained soil. We used a selected soil material (Figure 1) of similar, almost identical grain-size distribution, but of a different microstructure, which was characterised by the total shape index *ζ*_0*C*_ that expresses the variability of sphericity, angularity, and roughness Zięba [28] according to Parylak [32]. The method of determining this parameter is described in Equation (1).

(1) $$\zeta_{0C}=\frac{\zeta_{\Phi }+\zeta_{1-A}+\zeta_{1-I_{a}}}{3}\left[-\right]$$

where:

*ζ_Φ_*—sphericity index; *ζ*_1−*A*_—angularity index; *ζ*_1−*Ia*_—roughnes index.

In this case, the microstructure ranged from ideally spherical, smooth particles (artificial glass microbeads, *ζ*_0*C*_ = 1.00) to highly irregular and rough ones (fly ash, *ζ*_0*C*_ = 0.48) (Figure 1). 

One studied soil material was of a natural character (sandy silt from Krakowiany SK—Figure 1b) and three were of an anthropogenic character (glass microbeads GM—Figure 1a, sandy silt from Graniczna SG, Figure 1c, and fly ash FA—Figure 1d). Their detailed origin is as follows: GM—factory-produced 100% *glass microbeads*; SK—natural soil from Krakowiany, Lower Silesian Voivodeship, Poland; SG—granite processing waste obtained from Graniczna near Strzegom, Lower Silesian Voivodeship, Poland; FA—fly ash from hard coal combustion, wet storage—Łaziska Power Plant, Silesian Voivodeship, Poland.

An important boundary condition of the study was the differences in the parameter of sphericity in various fine-grained materials (Figure 1). Sphericity had the maximum value of 100% in the first material (GM), and it decreased gradually to 45% in the second (SK), 26% in the third (SG), and 27% in the fourth (FA). Another important parameter was angularity. The first material (GM) had almost zero angularity (the material was highly spherical, concave), but the angularity increased in the second material (SK) to 34%, in the third material (SG) by 9% to 43%, and in the fourth material (FA) by 19% to 62%. If we quantify changes in roughness of the studied soil materials, we observe zero roughness in GM an increase to almost 10% in SK, to 11% in SG, and to 22% in FA. This means that to evaluate the factors, the differences in the materials need to be substantial to be able to prove their influence on the changes in permeability coefficient in terms of engineering-geological environment permeability.

To be able to study the effect of shape characteristics on the changes in permeability coefficient, it was vital to comply with a boundary condition of having soil materials in the case studies with almost identical particle sizes (Table 1). 

Other important characteristics of soil materials in the case studies were total porosity, effective porosity, total shape index and specific surface area—see Table 1. Minimum total porosity of 0.27 was observed in the first anthropogenic soil (GM) at density index (I_D_) 90%, and maximum total porosity of 0.51 was reported in fly ash at density index 10%. Minimum effective porosity of 0.20 was observed in FA at density index 90%, and maximum effective porosity of 0.38 was reported in GM at density index of 10%. 

**Figure 2:**
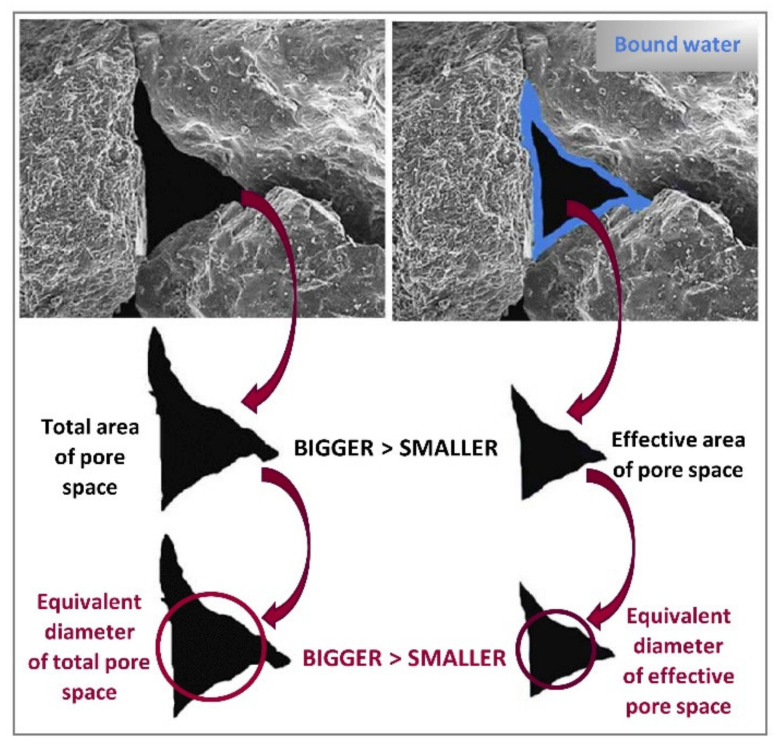
Pore space analysis based on SEM image.

An important boundary condition of permeability coefficient is soil porosity (Figure 2), in the form of total porosity [33], but much more important in this case is effective porosity [34,35]. The value of effective porosity is closely related to specific surface area which reflects the roughness of the particle surface and determines the soil capability to retain bound waters [36,37]. Which is why, the equivalent diameter of total pore space is bigger than the equivalent diameter of effective pore space (Figure 2).

**Figure 3:**
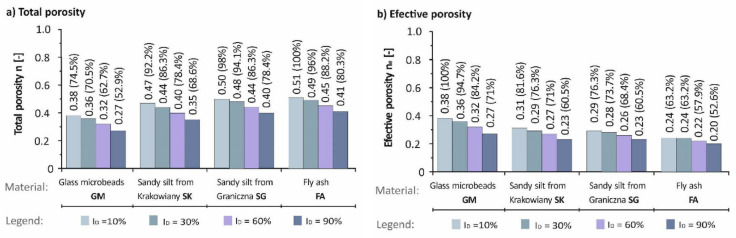
Porosity of the studied soil samples: (**a**) Total porosity, (**b**) Effective porosity.

This relationship manifests itself significantly in the varying values of total porosity (Figure 3a) and effective porosity (Figure 3b). Based on the increasing roughness from glass microbeads to fly ash in the four studied soil materials, it may be concluded that the smoothest surface of the first anthropogenic soil (GM) has analogous values of total and effective porosity. On the other hand, the roughest surface of the fourth soil material (FA) has a vast difference between the total and effective porosity caused by the roughness and a more pronounced zone of bound water around the grains. The values of total and effective porosity of the second (SK) and third soil material (SG) fall between the values of GM and FA. Thus, the dependence is visible in a gradual increase between the total and effective porosity of samples. 

### 2.2. Methods Used to Determine the Permeability Coefficient

**Figure 4:**
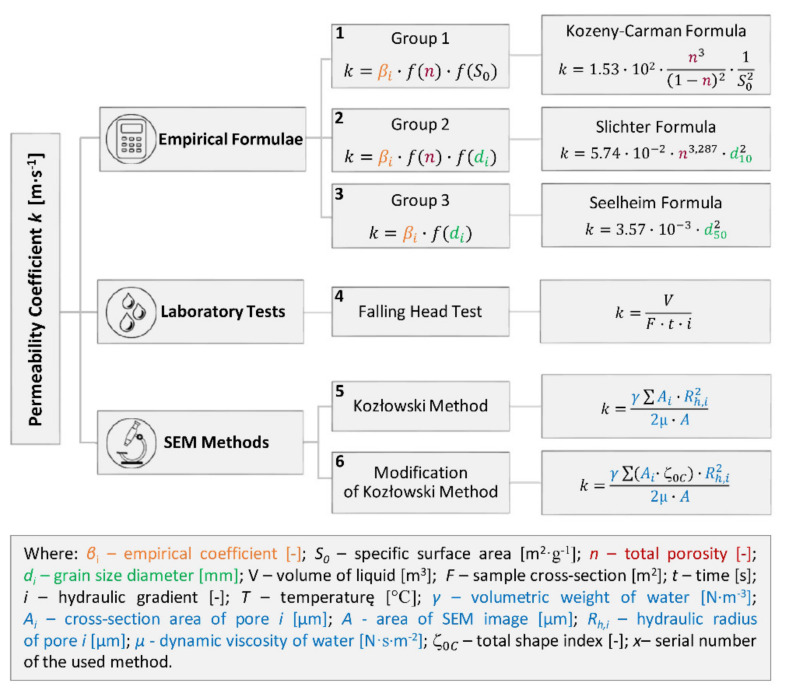
Methods of permeability coefficient determination applied (the differences between the formulae are marked in different colours).

For the purposes of this article, the permeability coefficient was determined using 6 methods, including laboratory tests and empirical formulae as well as innovative techniques for determining the permeability coefficient based on the analysis of scanning electron microscope images (Figure 4).

**Figure 5:**
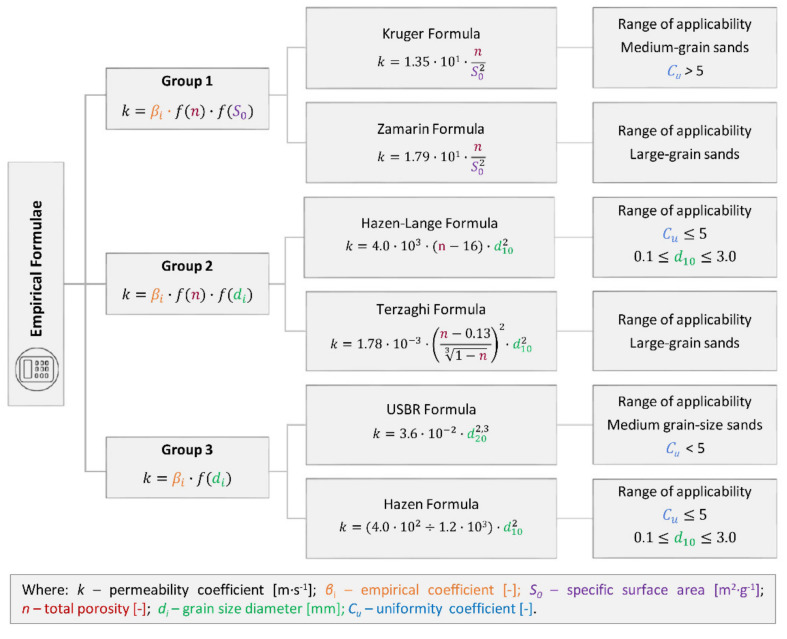
Other empirical formulae. The differences between the formulae are marked in colours: Kruger Formula [17,38], Hazen-Lange Formula [38,39], Hazen Formula [38,39], USBR Formula [17], Zamarin Formula [22,38,40], Terzaghi Formula [38,40].

The authors divided the empirical formulae available in the literature into three groups and selected one formula from each group that could be applied to the studied soils. The three applied methods of empirical formulae (Figure 4) must be understood in a wider context of all other methods summarized in Figure 5 which also presents ones whose boundary conditions are not suitable for fine-grained soils. This context (Figure 5) shows important analogies in the calculations for each group.

The first formula (Figure 4) falls in the group of empirical methods where equation take into account the function of porosity *f*(*n*) and the function of specific surface area *f*(*S*_0_), which reflects the effective porosity. The second formula falls in the second group, taking into account the function of porosity *f*(*n*) and the function of grain size diameter *f*(*d_i_*). The third formula from the third group accounts for the function of grain diameter *f*(*d_i_*) only. Each formula thus contains empirical coefficient *β_i_*. The permeability coefficient *k* was expressed in [m·s^−1^].

With regard to the range of applicability of individual formulae, the following equations were selected. From the first group we chose the empirical formula Kozeny-Carman based on the boundary condition of ranges of applicability-silts, sands, and gravelly sands [39,41]. From the second group, we selected Slichter Formula because of the range of applicability—0.01 mm ≤ d_10_ ≤ 5 mm [17]. From group 3, we chose the Seelheim Formula based on the range of applicability—sands, clay and elutriated chalk [22].

The fourth method (falling head test FHT) used to measure the permeability coefficient belonged to the group of laboratory tests [42]. The calculation of the permeability coefficient *k* took into account the amount of water flow *V* through the sample cross-section *F* in time *t* at given hydraulic gradient *i*.

The fifth and sixth methods were from the group of SEM methods. The fifth applied method (Kozłowski method—SEM K) is based on the analysis of scanning electron microscope SEM images [43]. The formula takes into account volumetric weight of water *γ*, dynamic viscosity of water *μ* (both at 10 °C), area of the SEM image *A* (concerning total porosity), cross-section area of pore *i* (*Ai*) and hydraulic radius of pore *i* (*Rh,_i_*). However, this method recognizes the total pore space area (not effective), so it does not reflect the influence of the microstructure of the soil particles.

Therefore, in the sixth method SEM K-Z, the authors modified Kozłowski’s method based on an empirical analysis of effective pore diameter with reference to the soil microstructure [28]. The total shape index (ζ_0*C*_) was introduced as a parameter reducing the value of pores cross-section area to obtain the effective pore space area. The same assumption was made for the determination of the effective porosity.

**Figure 6:**
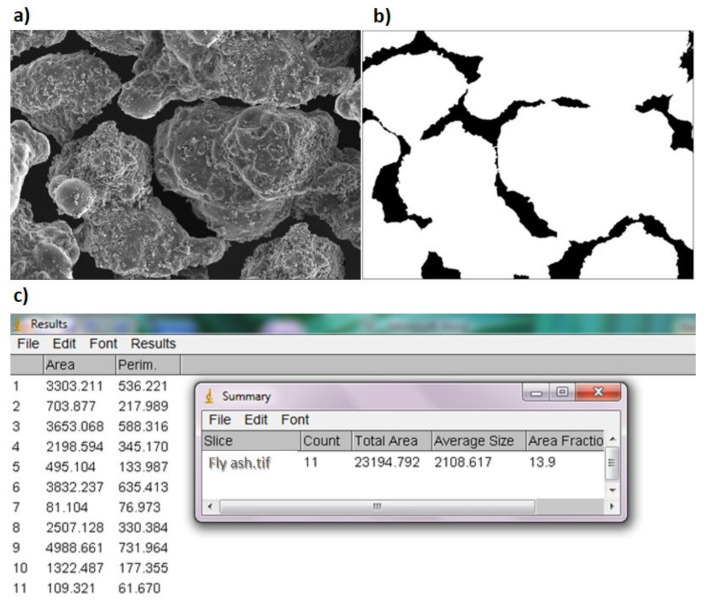
Analysis of the pore space of a single image using ImageJ software on example of fly ash: (**a**) output image; (**b**) image after transformations; (**c**) geometric pore parameters.

In both SEM methods, the permeability coefficient was determined on the basis of image analysis. Thirty SEM photographs of each variant (different soils with different density index) were analysed. The geometrical parameters of pore spaces were identified using ImageJ software (Figure 6). Based on this, the values of permeability coefficient were determined for each photograph. The obtained results were subjected to statistical analysis. For each variant, the arithmetic mean and standard deviation were determined for the significance level equal to 0.05. In addition, the coefficient of variation was calculated. When it exceeded 10%, extreme values of permeability coefficient were rejected and other results were averaged [44].

## 3. Results and Discussion

**Figure 7:**
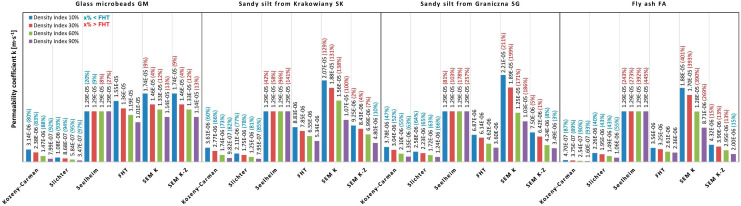
The values of permeability coefficient k obtained with different methods for different soils in relation to FHT.

Based on the results of the tests and calculations of the permeability coefficient (Figure 4), the evaluation of individual methods was performed. The results obtained from the laboratory tests (FHT) were taken as the reference values (Figure 7). In this method, the permeability coefficient was determined on the basis of the actual water flow through the soil samples.

The results imply that higher permeability values in the first soil material and a gradual decreasing trend from the second through the fourth soil material (Figure 7). This is explained by the gradual fall in the grain sphericity and an increase in grain angularity and roughness from the first to the fourth soil material. It is best seen using the reference method four and it may also be observed in method six. The remaining methods confirm the trend only partially, which relates to the methodological shortages described in the previous section. 

In principle, we can observe in the results that the first two empirical methods (Kozeny-Carman and Slichter) show lower permeability coefficients from 40% to 97% than the reference laboratory method FHT. The third empirical Seelheim Formula presents significantly higher values than the reference method, except for two values in the first anthropogenic soil material. The microscopic Kozlowski method SEM K mostly shows significantly higher values than the reference method (in the order of hundreds per cent), except for the first anthropogenic soil material (GM), where the values are higher by only 9 to 13%. The best compatibility was achieved using the sixth method (SEM K-Z), where the maximum difference with the reference method was below 15%, but the majority of the measured values had a difference of a single digit value (Figure 7).

**Figure 8:**
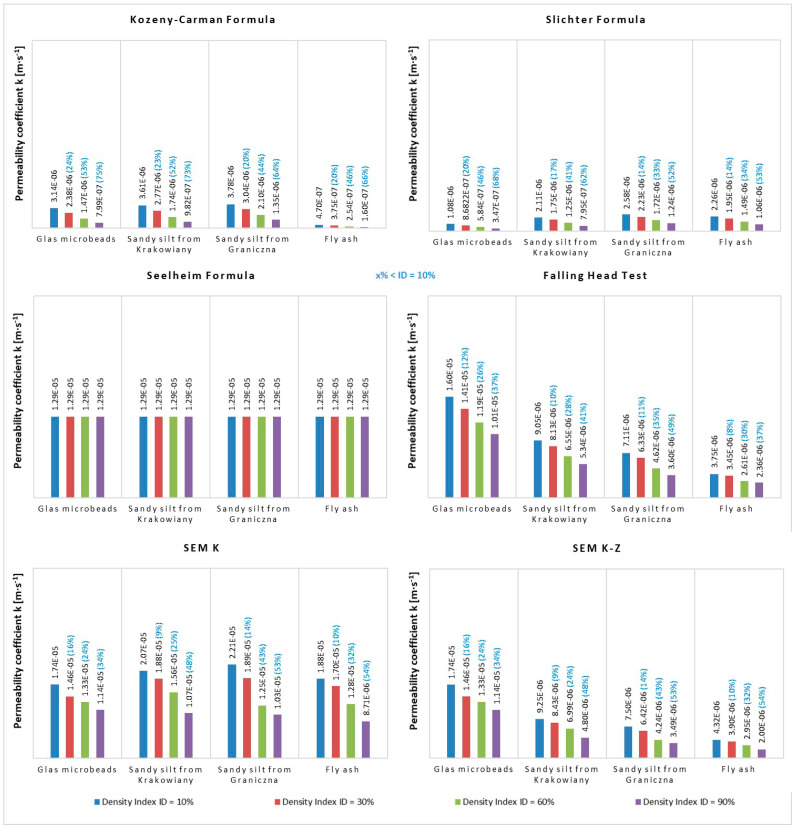
The values of permeability coefficient k obtained with the same method for different soils in relation to the density index I_D_.

If we evaluate the values of permeability coefficient based on the different methods applied (Figure 8), the following findings are achieved. The Kozeny-Carman Formula (Figure 8a) shows a decrease in density index in all studied materials from the most compacted state to the least. This is logical because more water permeates through looser and less compacted material than through more compacted one. The difference between the least compacted state (density index I_D_ = 10%) and the most compacted state (density index I_D_ = 90%) was 75% in GM. On the other hand, in SK the difference was 73% and the decreasing trend continued in other soil materials, i.e., 64% in SG and 66% in FA. 

A similar trend of decreasing values based on density index was also observed in the Slichter Formula (68%, 62%, 52%, 53%). In Seelheim Formula the trend differed. Density did not seem to play any role in the soil materials and the hydraulic permeability value stayed unchanged. In falling head test (fourth method) the trend of permeability coefficient was similar to the first and second empirical formulae, i.e., the values fell along with higher compaction, but the percentage difference between the first and last value was slightly smaller (37%, 41%, 49%, 37%) than in the first and second methods. In the microscopic methods the trend was similar in the fact that the permeability coefficient values fell from the least to the most compacted state. However, the differences in values of the four soil materials had a reversed course than in the previous case. This means that differences in values grew (34%, 48%, 53%, 54%). The fourth and fifth methods had analogous percentage differences between changes in the states of compaction.

The acquired results imply that the most suitable method out of the six applied to determine permeability coefficient was the fourth method of reference laboratory falling head test, as it reflected the changes in the state of compaction between the least and most compacted state. At the same time, it most closely reflected changes between concave grains of GM and most angular grains of FA. Fly ash had the best boundary conditions to achieve best state of compaction. Thus, GM had the worst boundary conditions to achieve the best state of compaction. All the facts are presented in the trend of permeability coefficient changes (Figure 8d).

The second most suitable method was the modified Kozlowski method (SEM K-Z) (Figure 8f). Similarly to the FHT, the modified Kozlowski method reflected the physical process of soil compaction in the gradual decrease in permeability coefficient from the least compacted to the most compacted state. In addition, the method reflected the shape and material character of the soil grains. More angular grains compact in a better manner than concave grains. Thus, permeability coefficient falls from more suitable to less suitable shapes in terms of compaction.

The third most useful was the Kozlowski method (SEM K). Although it reflected the process of gradual compaction of the different soil materials, the permeability coefficient values only partially accounted for the difference in grain shapes. Despite the fact that materials with more concave grains should have a higher permeability coefficient than materials with more angular grains (Figure 8e), this was only fully reflected in the modified Kozlowski method SEM K-Z (Figure 8f).

The fourth in terms of suitability was the Kozeny-Carman Formula (Figure 8a) and the fifth was Slichter Formula (Figure 8b). In case of both formulae, it may be stated that permeability coefficient fell along with a higher compaction state. However, similarly to the Kozlowski method, the differences between the grain shapes were not reflected in the permeability coefficient values, and the final values of permeability coefficient were even lower than in the more methodically optimal laboratory (SEM) methods. 

The last, i.e., the least suitable, was the Seelheim Formula (Figure 8c). This method took into account only the diameter d_50_ and did not reflect the process of gradual compaction of soil materials or the grain shape characteristic. Consequently, all soils have exactly the same permeability coefficient at each compaction, which is inconsistent with the actual water flow in the analysed materials.

The permeability coefficient is an important parameter in the assessment of soil environment, both in engineering geology and geotechnics. Thus, the research study aims to point at its importance in permeability assessment and in the choice of a suitable method of its determination. In the four case studies, the most suitable method was the reference laboratory FHT method. If permeability coefficient is not considered, wrong decisions may be taken in foundation engineering or other uses of engineering-geological environment. The study showed that the assessment of permeability coefficient using different methods led to different classifications of permeability, which may also result in wrong decisions. 

From the point of view of engineering practice, a correct description of the phenomenon of water flow in soil and a precise determination of the permeability coefficient that characterises it is particularly important. Therefore, it is crucial to choose an appropriate method to determine this parameter [18,19,45]. The research carried out in this paper proves that there are large discrepancies in the obtained results depending on the method used.

To date, numerous studies have been carried out showing significant differences between results obtained with different empirical formulae, as well as empirical formulae and laboratory and field studies [16,17,19,22,41,46,47,48] 

There have been few studies comparing different methods of determining permeability coefficient with consideration of particle shape characteristic and porosity. In their paper, Cabalar and Akbulut compared the values of permeability coefficient for sands with different gradation and shape, with the use of SEM images and simple indices classifying particle shape [18]. SEM images were used to demonstrate physical differences/similarities among the tested soils. Roundness and sphericity were estimated with a method proposed by Muszynski and Vitton [49]. Permeability coefficient was examined using a constant head method and predictive methods (Hazen, Kozeny-Carman, Terzaghi, Chapuis, Slitcher, USBR, NAVFAC, Alyamani and Sen, and Breyer). Slitcher and Terzaghi methods returned the best correlation with measured coefficient of permeability values, while Kozeny-Carman and NAVFAC approaches gave the worst correlation with measured values.

In the literature reviewed, the authors obtained results that cannot be compared with one another, nor can the tendency for individual empirical formulae to over- or underestimate results in comparison with laboratory methods be clearly identified. Few methods for determining permeability coefficient (both laboratory and empirical) can be applied to a wide range of grain sizes, hence global analysis and comparisons between studies are problematic. 

Microscopic methods may be considered as an alternative to empirical formulae for determining the permeability coefficient. Pioneer studies in this field were carried out by Kozłowski (SEM K method) [43], but they were limited to cohesive soils (clay). The modification of Kozlowski equation (SEM K-Z) extended the application of microscopic methods transitional soils. In practice, such approach to the problem allows to determine the permeability coefficient in a shorter time on many samples (irrespective of their grain size), also on small samples which are too tiny for laboratory tests.

In connection with engineering-geological and civil engineering practice, and especially with foundation engineering, soil classifications for different purposes or different properties are crucial. It is important to consider permeability [50] of foundation soils as one of the classification criteria. Next, we may ask whether it is more frequent to perceive permeable soils as more suitable foundation soils, or impermeable soils as suitable.

**Figure 9:**
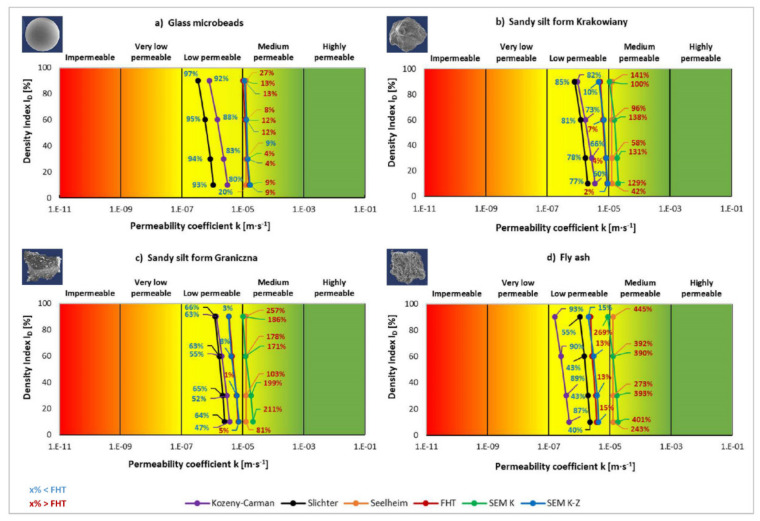
Purpose-made classification of engineering-geological environment permeability (suitable—green, conditionally suitable—yellow, unsuitable—red) based on permeability coefficient, where high permeability is perceived as a positive property of rock massive.

**Figure 10:**
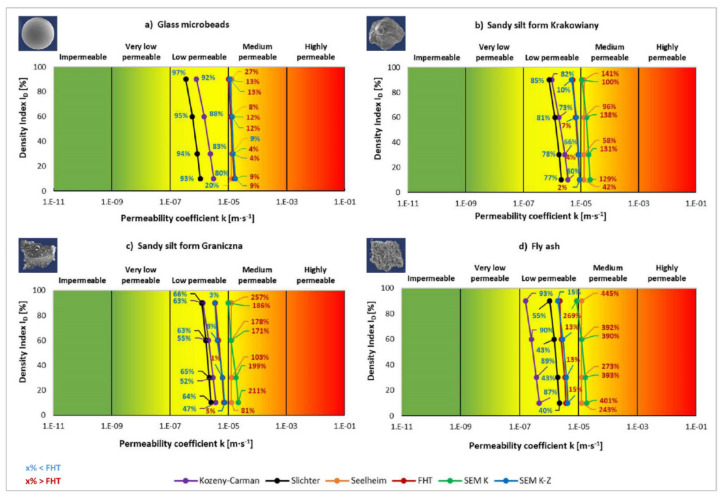
Purpose-made classification of engineering-geological environment permeability (suitable—green, conditionally suitable—yellow, unsuitable—red) based on permeability coefficient, where low permeability is perceived as a positive property of rock massive.

In fact, both assessment approaches are used, but more frequently permeable soil is considered more suitable. Research shows that permeable soils such as gravel and sandy soils are more suitable in terms of load-bearing capacity [51,52] and settlement [53,54] than fine-grained soils. The difference between the two approaches is thus seen in several engineering-geological properties, including permeability. Figure 9 shows permeable geological environment perceived as suitable foundation engineering soil. The classification of foundation engineering soils is grounded in European Standard ISO-14688-2:2004 [55]. On the other hand, Figure 10 shows impermeable geological environment as a suitable geological environment (less frequent approach). This approach is typical for engineering geology, geotechnics and foundation engineering [56,57,58,59]. This is due to the fact that the realised objective, i.e., a building, determines the suitability or unsuitability of certain properties of foundation soil, which is variable depending on the objective.

If we evaluate the determination of permeability coefficient, it proves to be a highly sensitive topic when one sample is considered suitable using one classification method and simultaneously unsuitable (or conditionally suitable) using another classification method. In terms of practicality, this may mean that we may select an inappropriate approach during foundation engineering or other interference with the geological environment.

It is more common to assess permeable geological environment as a suitable foundation engineering soil (Figure 9). Important parameters were values obtained using the reference falling head test (FHT), which shows more realistic values of permeability coefficient. In SK, SG, and FA (apart from GM), the permeability coefficient according to FHT was classified in the sector of conditionally suitable foundation engineering soils, i.e., with low permeable soil. On the contrary, FHT classified the anthropogenic soil material of glass microbeads in the green sector of suitable (medium permeable) foundation engineering soils. The results imply that the determination of permeability coefficient according to different methods leads to different classifications of foundation soils. In the four case studies, some methods classified the same sample into conditionally suitable or suitable foundation soils (Figure 9). The results close to the reference method FHT should be decisive. 

This type of classification is suitable for all types of structures, where permeable soils represent appropriate load-bearing foundation soil because of more fitting physical-mechanical properties in terms of load-bearing capacity. It corresponds to the fact that permeable soils have better capacity to drain water and thus are subjected to fewer changes in the volume, which brings more positive connections in load-bearing capacity and settlement. On the contrary, low permeable fine-grained soils, which are the subject of the four case studies, represent more problematic foundation soil (conditionally suitable). In particular, problems are encountered if they are found in soft or slushy consistencies. The anthropogenic soil material of glass microbeads is a certain exception as it is more permeable and thus conditionally suitable.

The second, less common approach is when impermeable engineering-geological environment is considered a suitable foundation soil for a given purpose. This way, the impermeable engineering-geological environment of fine-grained soils may be more suitable foundation soil. Therefore, sandy silt from Krakowiany, sandy silt from Graniczna, and fly ash are conditionally suitable foundation soils—see the yellow colour in Figure 10. All the values of these three studied soils (including the changes in density index) fell in this part of the classification graph using the reference falling head test. Only the anthropogenic soil of glass microbeads was classified as unsuitable because it was more permeable due to the grain shape. 

Using this opposite approach (Figure 10), the application of different methods led to different classifications. If a method is selected inappropriately, the soil may be classified based on the permeability coefficient as low permeable using FHT, but as medium permeable using another one. It may cause problems in engineering geology and foundation engineering.

## 4. Conclusions

The study points at the fact that the permeability of the engineering-geological environment perceived through permeability coefficient is an important scholarly topic with a number of boundary conditions that limit or enable its application in engineering geology, geotechnics, and foundation engineering.

The results show that grain sphericity, angularity, and roughness in fine-grained soils are very important parameters of foundation soils affecting the permeability expressed by permeability coefficient. Clearly, if we have soils of analogous grain-size distribution but different grain shape and roughness, the permeability (permeability coefficient) increases along with a rise in the sphericity and concaveness and a fall in grain angularity and roughness. Also, along with a rise in angularity (lower sphericity and concaveness) and roughness, the permeability decreases, manifesting in lower permeability coefficient. The difference in the permeability coefficient between the most spherical (least angular and rough) grains of glass microbeads was four-fold in comparison with the most angular and roughest fly ash. Moreover, the difference increases further, along with higher compaction, which means that at higher compaction, the differences in grain shapes are even more important. 

When evaluating the effect of compaction on the permeability coefficient values and thus on permeability of the selected fine-grained soil materials, it may be stated that the effect of compaction was studied using four states of compaction (density index 10%, 30%, 60%, and 90%). Using the laboratory method, we identified that there was a similar efficiency of compaction in the most angular and roughest FA, where the difference between the minimum and maximum compaction was 37%, which manifested itself in lower permeability coefficient values. Similar effects occurred in the most concave grains too (GM). In the two remaining materials, the difference between the minimum and maximum compaction states was 41% (SK) and 49% (SG). Using the modified microscopic method, we found that the efficiency of compaction increased gradually (34%, 48% 53%, 54%) along with changes in the grain shape and roughness from the least angular and least rough to the most angular and roughest shapes, which was demonstrated in a gradual decrease of the permeability coefficient and permeability.

Another assessment criterion in the study was the choice of methods in determining the permeability coefficient in selected fine-grained soils with analogous grain-size distributions. FHT was adopted as the reference method, because in this laboratory test the permeability coefficient was determined based on water flow through the soil. Using FHT, the permeability coefficient values reflected the differences in the soil material grain shape as well as states of compactness expressed as density index. Comparable results were achieved using the modified Kozlowski method, while using the four remaining approaches we observe a number of drawbacks described above.

Another aspect of the study assessment was to point at the differences in the permeability coefficient values on particular classifications of engineering-geological environment permeability when determining the suitability or unsuitability of rock/soil massive for a particular purpose. The results show that the influence is clear and it is vital to approach the choice of permeability coefficient assessment methods in a sensitive manner and take into account also the grain shape and roughness. Also, it is important to consider effective porosity and density index. This is because all these boundary conditions have specific influence on the purpose classification systems of engineering-geological environment permeability used in engineering geology, geotechnics, and foundation engineering. The results prove that all the boundary conditions may manifest themselves in altered classification category of suitable, conditionally suitable or unsuitable engineering-geological environment for a particular purpose. This may have negative consequences in foundation engineering as these purpose classifications are important tools in foundation engineering processes. The study proved that certain permeability of engineering-geological environment may constitute a suitable engineering-geological environment for one purpose, but conditionally suitable or unsuitable for another.

## Figures and Tables

**Table 1:** Input parameters of soils.

Soil Type	Density Index	Particle Size Diameter									Total Porosity	Effective Porosity	Total Shape Index	Specific Surface Area
	I_D_	d_10_	d_20_	d_30_	d_40_	d_50_	d_60_	d_70_	d_80_	d_90_	n	n_e_	*ζ*_0*C*_	S_0_
	[%]	[mm]	[mm]	[mm]	[mm]	[mm]	[mm]	[mm]	[mm]	[mm]	[–]	[–]	[–]	[m^2^·g^−1^]
Glass microbeads (GM)	10	0.021	0.027	0.033	0.043	0.060	0.071	0.091	0.120	0.229	0.38	0.38	1.0	0.268
	30	0.021	0.027	0.033	0.043	0.060	0.071	0.091	0.120	0.229	0.36	0.36	1.0	0.268
	60	0.021	0.027	0.033	0.043	0.060	0.071	0.091	0.120	0.229	0.32	0.32	1.0	0.268
	90	0.021	0.027	0.033	0.043	0.060	0.071	0.091	0.120	0.229	0.27	0.27	1.0	0.268
Sandy silt from Krakowiany (SK)	10	0.021	0.027	0.033	0.043	0.060	0.080	0.110	0.160	0.229	0.47	0.31	0.67	0.395
	30	0.021	0.027	0.033	0.043	0.060	0.080	0.110	0.160	0.229	0.44	0.29	0.67	0.395
	60	0.021	0.027	0.033	0.043	0.060	0.080	0.110	0.160	0.229	0.40	0.27	0.67	0.395
	90	0.021	0.027	0.033	0.043	0.060	0.080	0.110	0.160	0.229	0.35	0.23	0.67	0.395
Sandy silt from Graniczna (SG)	10	0.021	0.027	0.033	0.043	0.060	0.080	0.110	0.160	0.229	0.50	0.29	0.58	0.448
	30	0.021	0.027	0.033	0.043	0.060	0.080	0.110	0.160	0.229	0.48	0.28	0.58	0.448
	60	0.021	0.027	0.033	0.043	0.060	0.080	0.110	0.160	0.229	0.44	0.26	0.58	0.448
	90	0.021	0.027	0.033	0.043	0.060	0.080	0.110	0.160	0.229	0.40	0.23	0.58	0.448
Fly ash (FA)	10	0.019	0.027	0.033	0.043	0.060	0.080	0.110	0.160	0.229	0.51	0.24	0.48	1.340
	30	0.019	0.027	0.033	0.043	0.060	0.080	0.110	0.160	0.229	0.49	0.24	0.48	1.340
	60	0.019	0.027	0.033	0.043	0.060	0.080	0.110	0.160	0.229	0.45	0.22	0.48	1.340
	90	0.019	0.027	0.033	0.043	0.060	0.080	0.110	0.160	0.229	0.41	0.20	0.48	1.340

## Data Availability

The data that support the findings of this study are available from the corresponding author upon reasonable request.
